# Common institutional investors and the quality of management earnings forecasts—Empirical and machine learning evidences

**DOI:** 10.1371/journal.pone.0290126

**Published:** 2023-10-16

**Authors:** Shanshan Yang, Xiaohan Li, Zhenhua Jiang, Man Xiao

**Affiliations:** 1 Business School, Chengdu University, Chengdu, China; 2 Sichuan Provincial Housing Provident Fund Management Center, Chengdu, China; 3 Southwest Regional Air Traffic Management Bureau of Civil Aviation of China, Chengdu, China; 4 School of Management, Universiti Sains Malaysia, USM, Malaysia; University of Almeria: Universidad de Almeria, SPAIN

## Abstract

Based on the data of the Chinese A-share listed firms in China Shanghai and Shenzhen Stock Exchange from 2014 to 2021, this article explores the relationship between common institutional investors and the quality of management earnings forecasts. The study used the multiple linear regression model and empirically found that common institutional investors positively impact the precision of earnings forecasts. This article also uses graph neural networks to predict the precision of earnings forecasts. Our findings have shown that common institutional investors form external supervision over restricting management to release a wide width of earnings forecasts, which helps to improve the risk warning function of earnings forecasts and promote the sustainable development of information disclosure from management in the Chinese capital market. One of the marginal contributions of this paper is that it enriches the literature related to the economic consequences of common institutional shareholding. Then, the neural network method used to predict the quality of management forecasts enhances the research method of institutional investors and the behavior of management earnings forecasts. Thirdly, this paper calls for strengthening information sharing and circulation among institutional investors to reduce information asymmetry between investors and management.

## 1. Introduction

In 2020, a Chinese firm listed on the Shenzhen Stock Exchange of China disclosed that its earnings forecast range for FY2019 was 4 million yuan to 8 million yuan; later, the management narrowed the performance forecast range from 4 million yuan to 6 million yuan. Rendong Holdings (002647) disclosed its net profit range for FY2021 as a loss of 100 million to 200 million yuan, whose difference was too wide compared to the original estimated range and received a supervisory letter from the Shenzhen Stock Exchange. For particular motives, the shareholders and management strategically release a wider earnings forecast range based on meeting the minimum regulatory requirements to obscure the accurate judgment of investors, creditors, and customers on the firm’s performance [1]. This strategic disclosure behavior weakens the risk warning function of performance forecasts, causing a decline in the information content of performance forecasts and undermining the development of sustainable accounting information disclosure in the capital market [2,3].

In exploring the external factors influencing the quality of management earnings forecast, prior literature has made analysis and research mainly from analyst tracking, market competition [4], and exchange inquiry letters [5]. On the other hand, internal factors such as executive traits, compensation incentives, and shareholding structure [6] also have an important impact on earnings forecast. Institutional shareholding as a crucial external monitoring mechanism influences management voluntary disclosure [6,7].

With the upgrading of the professional asset management industry, coupled with the "do not put your eggs in the same basket" strategy of diversification, a unique ownership structure of common institutional investors in the industry has been formed, which refers to institutional shareholders holding large shares in competitive companies in the same industry and having a significant influence on company decisions. Institutional shareholders have a significant influence on corporate decisions [6,8–10]. Prior literature shows that shareholders influence management’s behavioral decisions, and managers are well aware of large shareholder incentives [11,12]. The sensitivity of executive compensation to performance gradually decreases when the industry is more institutionally co-owned [13]. The common institutional shareholders change the competitive preferences of the industry [14], and the management should be able to perceive such preference changes and adjust their behavioral decisions in time [15]. Studies related to common institutional shareholders focus on the following aspects to do further research on corporate competition [16–19], financing ability [20], corporate innovation [21], and corporate governance [22–24].

In recent years, scholars have gradually transitioned their research on institutional shareholding on performance preview disclosure from a single institution to an institutional network, generally based on the network linkage formed by multiple institutions investing in the same enterprise together [10]. As the information disclosure environment in China is semi-mandatory, there are specific research gaps on whether and how the unique shareholding structure of industry-shared institutional investors affects the quality of management earnings forecasts of China’s listed companies.

This paper’s marginal contributions and implications are: First, this paper examines the impact of common institutional investors on the quality of management earnings forecast, which enriches the literature related to the economic consequences of common institutional shareholding. Secondly, we introduce the non-controlling large shareholder exit threat variable (*NET*) and use the neural network method to predict the precision of management forecast, enhancing the research method of institutional investors and the behavior of management earnings forecast. Finally, it provides empirical and machine learning support for information sharing and collaborative supervision among industry associations, listed companies, and investors, which helps to further standardize the corporate governance structure and promote the high-quality development of listed companies.

## 2. Theory background and hypothesis development

Earnings forecasts are forward-looking, convey macroeconomic conditions of the industry in which the firm is located, and contain proprietary information about the firm’s operating conditions, market position, and risk information [25,26]. Competitors commonly obtain proprietary information from the disclosure of earnings forecasts to adjust their competitive strategies, adversely affecting the firm’s competitive position in the market. Therefore, the cost of proprietary information becomes one of the main limitations of management earnings forecasts. With the gradual alleviation of the degree of competition in the product market due to common institutional investors’ shareholdings in the same industry, management is less concerned that the proprietary information conveyed in the disclosure will be used by competitors to gain excess market share or profits, thus relaxing the restrictions on full disclosure, provided that the cost of proprietary information is low. The benefits remain unchanged [6]. Second, regulators require and encourage complete and accurate management earnings forecasts; the potential compliance costs create a positive incentive for managers to provide high-quality earnings forecasts to the public. Third, in shareholder-initiated governance proposals, institutional investors can vote against incompetent management or even have incompetent executives ousted outright [24]. Management has the incentive to consider the competitive preferences of shared institutional investors to avoid the risk of stepping down and to improve the quality of management earnings forecasts. Based on this, hypothesis H1 is proposed:

H1: Common institutional investors significantly enhance the precision of management earnings forecasts.

Based on the above research, this paper will further explore predicting the quality of listed firms’ earnings forecasts, which makes this paper more practically relevant. Institutional blockholders can govern the firms through the threat of “exit”-selling the holding shares when managers underperform [8]. Mutual funds react strongly to large shareholders’ exits, leading to correlated exits that enhance corporate governance [27]. In the emerging capital market, the agent problem mainly manifests in the contradiction between the controlling and other shareholders. It is not uncommon for the controlling shareholders to encroach on the interests of other shareholders. Compared to small shareholders, non-controlling large shareholders possess many shares and specific professional skills. When intervention is ineffective, non-controlling large shareholders often release the “exit” threat as a bargaining chip with controlling shareholders. The exit threat is vital for large shareholders to achieve their governance goals [8].

With the development of machine learning technology, the application of machine learning algorithms in the securities market mainly includes the following aspects. First, analyze and predict the price fluctuation of the securities market. For example, the classical artificial intelligence support vector machine [28], neural network [29], and long-term and short-term memory networks [30] have been applied to predict stock market fluctuations. Second, analyze the effectiveness of stock evaluation indicators. For example, genetic algorithms [31], intelligent computing [32], deep learning [33], and other algorithms can effectively select and evaluate stock evaluation indicators. Third, Simulation analysis of the mechanism in the securities market, using intelligent algorithms to explain and express the stock market momentum effect [34], herding effect [35], irrational factors [36], and other abnormal phenomena. As the development direction of artificial intelligence, the deep learning model has the ability of distributed processing and can continuously learn and evolve through updating the weights in the algorithm to solve the problem of nonlinear data processing. Therefore, we will use deep learning technology to build the model to complete the prediction of the precision of management earnings forecasts for listed firms.

## 3. Model construction

### 3.1. Data

We selected A-share listed firms in the Shanghai Stock Exchange and Shenzhen Stock Exchange of China from 2014 to 2021. The Chinese Ministry of Finance (MOF) revised and introduced nine standards in 2014, including the basic accounting standard, the principles of long-term equity investment (CAS 2), and financial instruments (CAS 22). For the sake of robustness, 2014 is selected as the starting year of the sample. We screened the samples according to the following principles: (1) exclude the financial and insurance industries; (2) exclude the ST firms; ST refers to the special treatment carried out by listed firms with unusual financial positions. (3) exclude the samples in the year of IPO; (4) exclude the samples with missing main variables in the sample period. All continuous variables are winsored at the 1% level to mitigate the effect of the ultimate value. The data on management earnings forecast used in this paper are obtained from the WIND database; data on common institutional investors, corporate finance, and corporate governance are obtained from the CSMAR database. The descriptive statistics, correlation analysis, and multiple regression analysis of the main variables in this paper are processed using STATA 17.0 and Python3.

### 3.2 Key measures

1. Quality of earnings forecast (*PRECS*). In this paper, we use the precision of earnings forecast as a proxy for the quality of earnings forecast [37], defined as the width range of management earnings forecast. When the value is zero, the forecast precision is highest. The forecast precision is calculated as the difference between the forecasts’ lower and upper limits, divided by the absolute value of the estimates’ mean [1].
2. Common institutional investors. Drawing on existing literature [6,20,24,38,39], we use quarterly firm data and retain institutional investors with shareholdings of 5% or more (including 5%). Suppose institutional investors hold at least 5% shares in two or more other firms in the same quarter in the same industry. In that case, this indicates the existence of common institutional investors. In this paper, we construct indicators of the shareholding of common institutional investors in four dimensions: the dummy variable *Coz_dum* is used to indicate the existence of common institutional investors; the variable *Coz_num* is used to indicate the number of common institutional investors; the variable *Coz_degree* is used to indicate the degree of connections of common institutional investors; the variable *Coz_rate* is used to represent the percentage of shares held by institutional investors. Second, the threshold of 5% is chosen because 5% is the threshold for significant shareholding based on Chinese securities laws and regulations.
3. Control variables. Based on the existing literature [4–6,37,40], the following variables are selected as control variables in this paper, specifically: firm size (*SIZE*), leverage ratio (*LEV*), sales growth (*GSALES*), profitability (*ROA*), cash level (*OCF*), dual role (*DUAL*), board size (*BRD*), analyst following (*ANA*), management shareholding ratio (*MGTSHR*), executive compensation (*PAY*), Big 4 audit (*BIG4*), and voluntary disclosure (*VOL*). The specific variables are defined in Table 1.
4. Non-controlling Large Shareholder Exit Threat (*NET*). Dou et al. measure that the blockholder’s exit threat is mainly affected by stock liquidity and competition among large shareholders, and they multiply stock liquidity and large shareholder competition as proxy variables for the blockholder exit threat [41]. The more liquid the stock is, the more intense the competition among large shareholders is, and the blockholder exit threat of the firm is higher [42].

**Table 1:** Definitions of variables.

Variable	Variable definition
*PRECS*	Quality of earnings forecasts, calculated as (Upper limit of earnings forecast range- lower limit of earnings forecast range) / absolute value of upper and lower limits
*Coz_dum*	Existence of common institutional investors. Dummy variable, quarterly, takes the value of 1 if the listed company is held by common institutional investors in the year, otherwise it takes the value of 0.
*Coz_num*	The number common institutional investors for each firm, then take the average and plus 1 to take the logarithm.
*Coz_degree*	The degree of connection of institutional investors. The average number of firms in the same industry held by all co-owned institutional investors, plus 1 to take the logarithm.
*Coz_rate*	The sum of the quarterly shareholding ratios of common institutional investors, averaged over the year.
*INSOWN*	The sum of shares held by institutional investors divided by the total number of issued shares is calculated by taking the logarithmic value.
*BIGOWN1*	Shareholding ratio of the largest shareholder as disclosed by the listed company
*SIZE*	Natural logarithm of the company’s total assets at the end of the year
*LEV*	Year-end gearing ratio
*GSALES*	sales growth rate
*ROA*	Profitability, equals to total net asset margin
*OCF*	Net operating cash flow divided by year-end total assets Normalized
*DUAL*	Dummy variable, takes the value of 1 if the chairman and CEO are the same person; otherwise, 0
*BRD*	Board Size, calculated as total number of board seats, logarithmically
*ANA*	The number of analyst teams that have followed the company during the year, plus 1, taking the natural logarithm
*BIG4*	Dummy variable for auditors. If the auditing firm belongs to the Big 4, take the value of 1; otherwise, 0
*MGTSHR*	Management shareholding ratio
*PAY*	Executive Compensation, calculated as natural logarithm of the average compensation of the top three executives
*VOL*	dummy variables for firms that have disclosed their earnings forecasts by mandatory and voluntary release. If it is voluntary disclosure, it takes the value of 1; otherwise, it takes the value of 0.
*NET*	Large shareholder Exit Threat Cross product of stock liquidity (SL) and the degree of competition from non-controlling majority shareholders (BHC)
*Year* FE	Year dummy variable
*Industry* FE	Industry dummy variables

In this paper, we refer to Dou et al.’s method and multiply stock liquidity (*SL*) and the degree of large shareholder competition (*BHC*) as the proxy variable for the exit threat of non-controlling large shareholders (*NET*) [41]. Drawing on Dou’s method, the average daily stock turnover rate of tradable stocks is used as a proxy variable for liquidity (*SL*). When the liquidity of a firm’s stock is high, it is easier for non-controlling large shareholders to exit, and stock liquidity increases the role of exit threat for non-controlling large shareholders. Since this paper mainly examines the exit threat role of non-controlling large shareholders, the econometric model for *BHC* is modified based on Dou et al. as follows:


$${BHC}_{it}=\sum_{K=1}^{N}\left(\frac{{NCLS}_{k,i,t}}{{SSBH}_{i,t}}\right)$$ (1)

Where BHC_*i*,*t*_ is the degree of competition of non-controlling large shareholders in year *t* of firm *i*, NCLS _*k*,*i*,*t*_ is the shareholding ratio of the *k*_th_ non-controlling major shareholder in year *t* of firm *i*, and *SSBH*_*i*,*t*_ is the sum of shareholding ratios of all major shareholders in year *t* of firm *i*. All the shareholding ratios here refer to the proportion of tradable shares. Therefore, a larger *BHC*_*i*,*t*_ indicates a higher degree of competition among non-controlling large shareholders. Finally, the econometric model of *NET* is constructed as follows:

$${NET}_{it}={SL}_{it}*{BHC}_{it}$$ (2)

### 3.3 Model and methodology

To verify the relationship between co-owned institutional investors and the precision of management earnings forecast, we construct the following model for OLS regression [3,6].


$$PRECS_{it}=\beta_{0}+\beta_{1}Coz_{it}+\gamma Controls_{it}+\sum_{t}Year_{t}FE+\sum_{j}Industry_{j}\mathrm{FE}+\varepsilon_{it}$$ (3)

Suppose the regression coefficient *β*_*1*_ of common institutional investors (Coz_*i*,*t*_) is significantly negative; In that case, it indicates that common institutional investors narrow the width of the management earnings forecast and improve the forecasts’ precision. Controls_*i*,*t*_ is a set of control variables, andε_*it*_ is an error term. The heteroskedasticity-robust standard error clustered at the firm level is used to ensure the robustness of the model [43]; the year (Year FE) and industry (Industry FE) effects are controlled to address the omitted variables that do not change over time.

**Fig 1:**
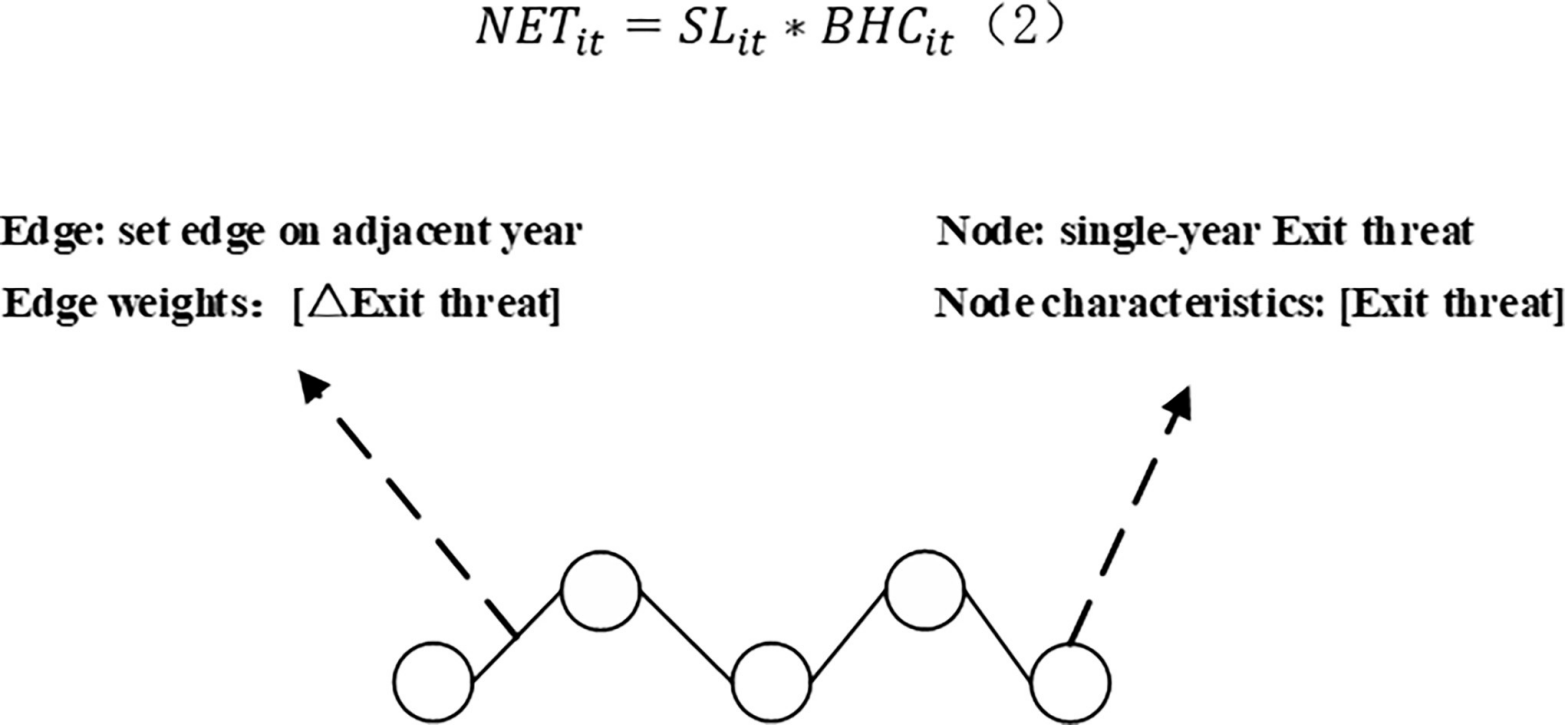
Exit threat variable graph embedding method.

In this study, the exit threat variable (*NET*) was introduced to further explore the forecasts precision. In order to fully interpret the trend of *NET* changes, this paper introduces the graph time series [28] embedding method to construct a graph with exit threat variables as a continuous time series. Embedding is shown in Fig 1; node characteristics are exit threat variables for the corresponding year, adjacent nodes are adjacent year nodes, adjacent years set edges, and edge weights are the magnitude of threat variable changes in adjacent years.

**Fig 2:**
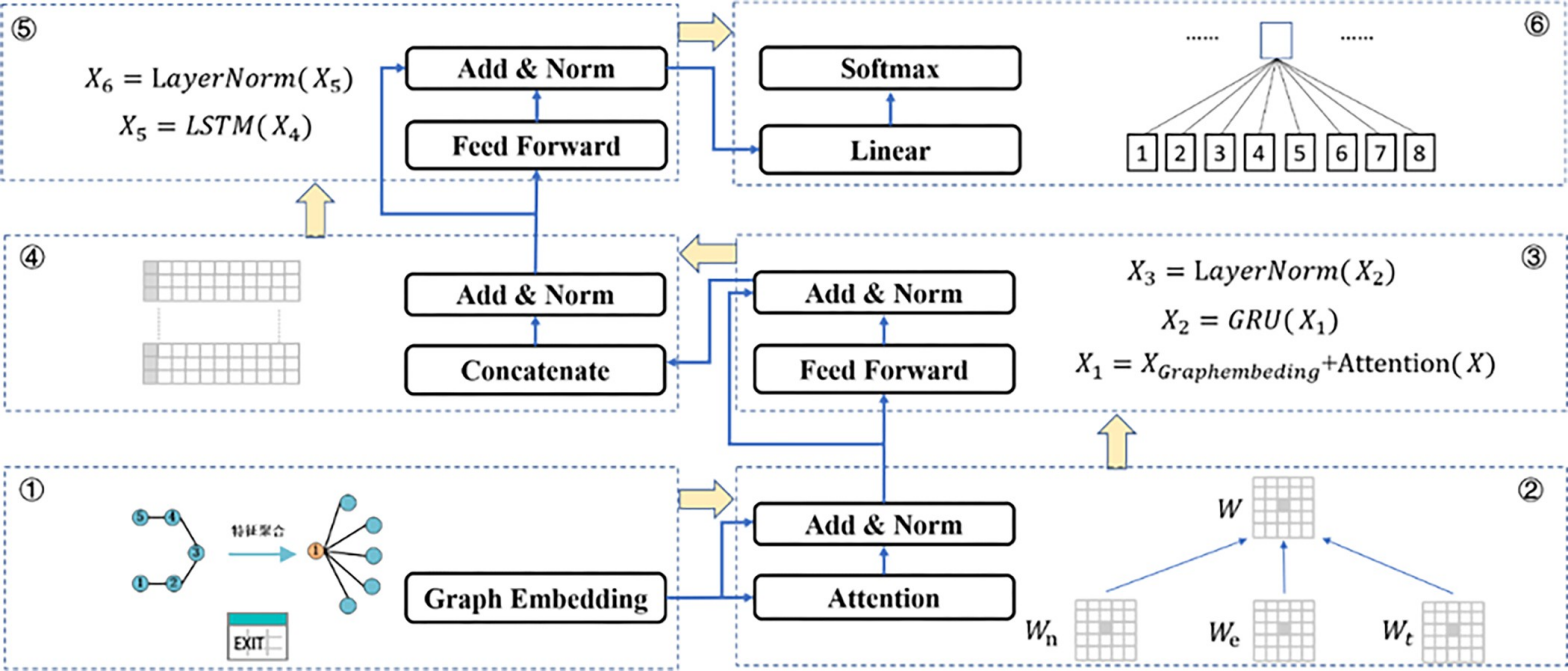
Flow chart of graph neural network for predicting the quality of forecasts.


Fig 2 presents the construction of the exit threat variable (*NET*) by graph embedding and the prediction of forecasts’ precision completed with a neural network. In Fig 2, part ① first constructs the exit threat variable graph data based on the method in Fig 1. Part ② then introduces the attention mechanism, and the node and edge weight matrices are shown in Eqs (4) and (5), respectively.

$$W_{N}=Linear(f_{N})=f_{N}w_{n}$$ (4)

$$W_{E}=Linear(f_{E})=f_{E}w_{e}$$ (5)

The attention mechanism is shown in Eq (6). *d_k_* is the dimension of the vectors *w_n_* and *w_e_*. Vector multiplication will increase the dimension. Therefore, to make the attention matrix have the characteristics of standard normal distribution, the $\sqrt{d_{k}}$ is introduced into the formula to process the matrix.


$$W_{a}=Softmax(NET/\sqrt{d_{k}})$$ (6)

In order to avoid the gradient explosion and disappearance problem, the residual connection is introduced to add the weight matrix and features directly as the hidden layer input, as shown in Eqs (7) and (8).


$$W=W_{N}W_{E}$$ (7)

$$W_{a}=W_{a}+W$$ (8)

GRU is used to further extract the features to form the feature vector matrix, and some feature information is discarded by the recurrent neural network forgetting gate. In contrast, the input gate adds new information to the node features and performs the state layer normalization process, as shown in Eqs (9) and (10).


$$W_{a}=GRU(W_{a})$$ (9)

$$W_{a}=LayerNorm(W_{a})$$ (10)

The transformed *NET* concatenate the variables in Table 1 to form a matrix, and according to the matrix features, the LSTM is introduced for binary classification to predict the forecasts precision. Prediction is achieved using the activation function *σ*. Finally, the whole convolutional embedding process is completed by Eq (11).


$$O_{t}=\sigma (Linear(LSTM(W_{a}^{’})))$$ (11)

The weight matrix is continuously updated through the aggregation update process. The model uses the cross-entropy loss function to complete the training process, and the model predicts the quality of earnings forecast of listed companies by dichotomizing the variable of *PRECS*.

## 4. Empirical results and analysis

### 4.1 Descriptive statistics

According to the descriptive statistics in Table 2, the mean value of *PRECS* in the sample firms is 0.2258, the standard deviation is 0.2153, and the maximum and minimum values are 1.3939 and 0.0000, respectively. It indicates that most firms have a narrow width of earnings forecasts and a high quality of management earnings forecast. 9.28% of the firms have common institutional investors. A firm has roughly one common institutional investor on average (the average value of *Coz_num* is 0.0672); the average value of the common institutional investor shareholding ratio (*Coz_rate*) is 2.00%, and the maximum value is 56.33%.

**Table 2:** Descriptive statistics of key variables.

Variable	N	Mean	p50	Min	Max	SD
*Coz_dum*	8,425	0.0928	0.0000	-	-	0.2902
*Coz_num*	8,425	0.0672	0.0000	0.0000	1.0986	0.2125
*Coz_degree*	8,425	0.1362	0.0000	0.0000	2.0794	0.4397
*Coz_rate*	8,425	0.0200	0.0000	0.0000	0.5633	0.0798
*PRECS*	8,425	0.2258	0.1754	0.0000	1.3939	0.2153
*INSOWN*	8,425	0.3884	0.3934	0.0094	0.8983	0.2414
*BIGOWN1*	8,425	0.3155	0.2972	0.0843	0.7121	0.1366
*SIZE*	8,425	22.2995	22.1349	20.2790	26.3883	1.1682
*LEV*	8,425	0.4053	0.3982	0.0634	0.8627	0.1932
*GSALES*	8,425	0.2581	0.1684	-0.4670	2.6731	0.4648
*ROA*	8,425	0.0484	0.0452	-0.1639	0.2325	0.0640
*OCF*	8,425	0.0510	0.0478	-0.1216	0.2479	0.0669
*DUAL*	8,425	0.3350	0.0000	-	-	0.4720
*BRD*	8,425	2.2728	2.3026	1.6094	2.8904	0.2484
*BIG4*	8,425	0.0402	0.0000	-	-	0.1965
*MGTSHR*	8,425	0.0947	0.0120	0.0000	0.5846	0.1466
*PAY*	8,425	13.4687	13.4240	11.9531	15.6390	0.6993
*ANA*	8,425	1.9477	1.9459	0.0000	3.9120	0.9306
*VOL*	8,425	0.3967	0.0000	-	-	0.4892

### 4.2 Pearson correlation coefficients matrix

Table 3 presents the Pearson correlation coefficients matrix of the main variables. There is a significant correlation between the indicators of independent variables (*Coz_dum*, *Coz_num*, *Coz_degree*, *Coz_rate*) and *PRECS*, which are all significantly negative at the 1% level. The correlation between common shareholders and higher *PRECS* preliminarily validates the research hypothesis in Section 2. In addition, there is a significant correlation between the control variables and *PRECS*. The correlation coefficient between no variables exceeds 0.5, and the value of each correlation coefficient is relatively small. There is no severe multicollinearity between the variables of the model.

**Table 3:** Pearson correlation coefficients matrix.

	*Coz_dum*	*Coz_num*	*Coz_degree*	*Coz_rate*	*PRECS*	*INSOWN*	*BIGOWN1*
*Coz_dum*	1						
*Coz_num*	0.987***	1					
*Coz_degree*	0.971***	0.978***	1				
*Coz_rate*	0.791***	0.815***	0.829***	1			
*PRECS*	-0.073***	-0.073***	-0.070***	-0.048***	1		
*INSOWN*	0.276***	0.285***	0.286***	0.285***	-0.082***	1	
*BIGOWN1*	0.061***	0.062***	0.070***	0.168***	-0.0130	0.493***	1
*SIZE*	0.338***	0.348***	0.351***	0.313***	-0.106***	0.474***	0.203***
*LEV*	0.129***	0.132***	0.137***	0.143***	0.0110	0.255***	0.110***
*GSALES*	-0.028***	-0.028***	-0.035***	-0.037***	-0.164***	-0.00100	-0.023***
*ROA*	0.018**	0.016*	0.00900	-0.035***	-0.233***	0.054***	0.079***
*OCF*	0.085***	0.085***	0.084***	0.048***	-0.153***	0.129***	0.108***
*DUAL*	-0.077***	-0.078***	-0.085***	-0.105***	0.00200	-0.205***	-0.062***
*BRD*	0.148***	0.158***	0.157***	0.157***	-0.0140	0.224***	0.016*
*Big4*	0.209***	0.215***	0.223***	0.195***	-0.036***	0.252***	0.142***
*MGTSHR*	-0.130***	-0.132***	-0.137***	-0.140***	-0.00900	-0.536***	-0.043***
*PAY*	0.202***	0.202***	0.199***	0.133***	-0.104***	0.213***	-0.0130
*ANA*	0.156***	0.154***	0.145***	0.071***	-0.162***	0.164***	0.0110
*VOL*	-0.075***	-0.078***	-0.077***	-0.078***	-0.033***	-0.065***	0.055***
	*SIZE*	*LEV*	*GSALES*	*ROA*	*OCF*	*DUAL*	*BRD*
*SIZE*	1						
*LEV*	0.568***	1					
*GSALES*	-0.00600	0.021**	1				
*ROA*	-0.081***	-0.380***	0.226***	1			
*OCF*	0.038***	-0.195***	0	0.466***	1		
*DUAL*	-0.173***	-0.112***	0.043***	0.041***	-0.020**	1	
*BRD*	0.246***	0.174***	0.00200	-0.084***	0.018**	-0.155***	1
*Big4*	0.314***	0.105***	-0.0130	0.028***	0.088***	-0.050***	0.093***
*MGTSHR*	-0.333***	-0.239***	0.065***	0.115***	-0.017*	0.486***	-0.200***
*PAY*	0.447***	0.147***	0.026***	0.177***	0.162***	0.00700	0.060***
*ANA TEAM*	0.294***	-0.015*	0.082***	0.356***	0.201***	0.026***	0.0110
*VOL*	-0.162***	-0.221***	-0.111***	0.223***	0.055***	0.031***	-0.090***
	*BIG4*	*MGTSHR*	*PAY*	*ANA*	*VOL*		
*BIG4*	1						
*MGTSHR*	-0.106***	1					
*PAY*	0.256***	-0.094***	1				
*ANA TEAM*	0.132***	0.017*	0.304***	1			
*VOL*	-0.034***	0.115***	-0.038***	0.124***	1		

### 4.3 Results

Table 4 presents the results of the empirical test of common institutional investors and the quality of performance forecasts. The results in columns (1)-(4) do not consider control variables. The results in columns (5)-(8) are added to each control variable. According to columns (5)-(8), common institutional investors (Coz) are positively correlated with performance forecast accuracy (PRECS). The estimated coefficients of *Coz_dum*, *Coz_num*, *Coz_degree*, and *Coz_rate* are -0.0172 (t = -1.9772), -0.0219 (t = -1.8008), -0.0121 (t = -2.0226), and -0.0821 (t = -2.2928), respectively, and are at the 5%, 10%, 5%, and 5% statistical levels of significance. It indicates that the common institutional investors narrowed the width range of management earnings forecast on average and improved the precision of management forecasts, as verified by H1. The finding is consistent with prior studies that common ownership increases in disclosure [6,44–46].

**Table 4:** Common institutional investors and the precision of earnings forecasts.

	(1)	(2)	(3)	(4)	(5)	(6)	(7)	(8)
	*PRECS*	*PRECS*	*PRECS*	*PRECS*	*PRECS*	*PRECS*	*PRECS*	*PRECS*
*Coz_dum*	-0.0395***				-0.0172**			
	(-4.6238)				(-1.9772)			
*Coz_num*		-0.0536***				-0.0219*		
		(-4.5946)				(-1.8008)		
*Coz_degree*			-0.0256***				-0.0121**	
			(-4.4191)				(-2.0226)	
*Coz_rate*				-0.1057***				-0.0821**
				(-2.8337)				(-2.2928)
*INSOWN*					-0.0610***	-0.0611***	-0.0608***	-0.0612***
					(-4.1564)	(-4.1646)	(-4.1405)	(-4.1589)
*BIGOWN1*					0.0625***	0.0626***	0.0625***	0.0676***
					(2.6764)	(2.6828)	(2.6749)	(2.8815)
*SIZE*					-0.0098***	-0.0098***	-0.0097***	-0.0094***
					(-3.0296)	(-3.0287)	(-2.9913)	(-2.9116)
*LEV*					-0.0281	-0.0281	-0.0283	-0.0288
					(-1.4886)	(-1.4897)	(-1.4996)	(-1.5248)
*GSALES*					-0.0528***	-0.0527***	-0.0529***	-0.0528***
					(-9.6828)	(-9.6795)	(-9.6978)	(-9.6982)
*ROA*					-0.5149***	-0.5146***	-0.5150***	-0.5159***
					(-9.6514)	(-9.6435)	(-9.6516)	(-9.6611)
*OCF*					-0.1579***	-0.1582***	-0.1576***	-0.1587***
					(-3.6651)	(-3.6712)	(-3.6588)	(-3.6890)
*DUAL*					0.0045	0.0045	0.0045	0.0042
					(0.7581)	(0.7618)	(0.7527)	(0.7056)
*BRD*					-0.0051	-0.0051	-0.0050	-0.0047
					(-0.4801)	(-0.4779)	(-0.4688)	(-0.4414)
*BIG4*					0.0031	0.0031	0.0035	0.0028
					(0.2064)	(0.2005)	(0.2308)	(0.1862)
*MGTSHR*					-0.0645***	-0.0645***	-0.0643***	-0.0647***
					(-2.9554)	(-2.9568)	(-2.9490)	(-2.9660)
*PAY*					0.0145***	0.0145***	0.0146***	0.0146***
					(3.2510)	(3.2395)	(3.2562)	(3.2647)
*ANA*					-0.0159***	-0.0159***	-0.0159***	-0.0162***
					(-5.4572)	(-5.4691)	(-5.4857)	(-5.5922)
*VOL*					-0.0233***	-0.0233***	-0.0234***	-0.0235***
					(-4.7301)	(-4.7304)	(-4.7392)	(-4.7693)
_cons	0.2916***	0.2915***	0.2914***	0.2917***	0.4294***	0.4302***	0.4264***	0.4191***
	(8.3817)	(8.3798)	(8.3664)	(8.3771)	(4.6812)	(4.6816)	(4.6381)	(4.5691)
*Year* FE	Yes	Yes	Yes	Yes	Yes	Yes	Yes	Yes
*Industry* FE	Yes	Yes	Yes	Yes	Yes	Yes	Yes	Yes
*N*	8425	8425	8425	8425	8425	8425	8425	8425
r2_a	0.0290	0.0289	0.0289	0.0278	0.1061	0.1060	0.1062	0.1064
Mean VIF	3.78	3.78	3.78	3.78	3.06	3.07	3.07	3.06

Note: T-values calculated from heteroskedasticity-robust standard errors and clustered at the firm level are in parentheses

(* *p* < 0.1

** *p* < 0.05

*** *p* < 0.01).

**Fig 3:**
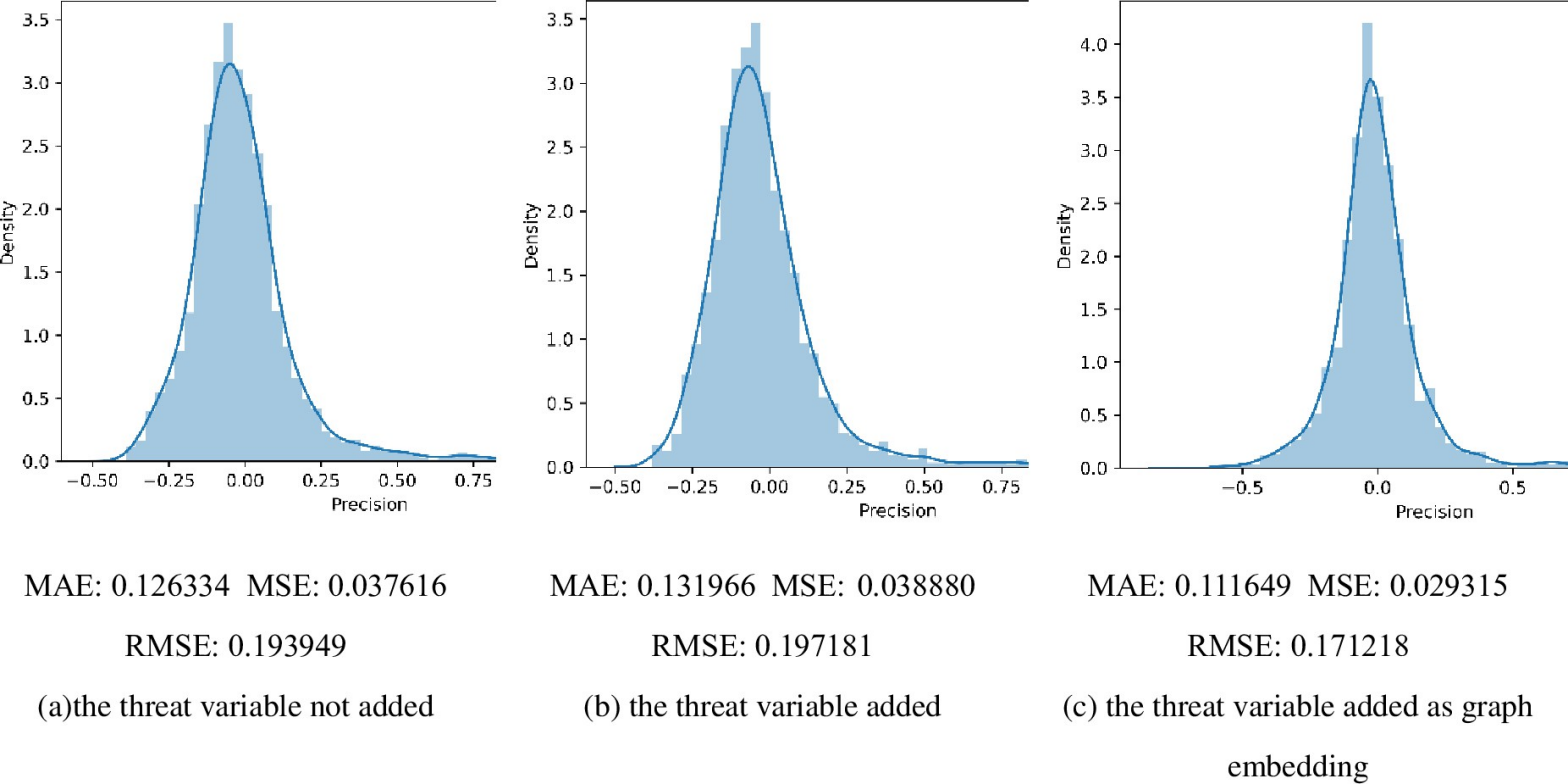
Results for the precision of earnings forecasts.

Fig 3 presents the results of the precision of earnings forecast using deep learning techniques. Furthermore, Fig 3 presents the test sample with the density profile of the difference between the predicted and actual results. Fig 3(A) shows the prediction results of the LSTM method without introducing the exit threat variable (*NET*). At the same time, Fig 3(B) presents the prediction results of the LSTM method with the introduction of the threat variable (*NET*) but with the threat variable as a class of features only. Fig 3(C), on the other hand, shows the prediction results using the embedding method of this paper to represent the threat exit variables as shown in the figure. The results from the density curves and the MAE, MSE, and RMSE metrics can be seen. The graph embedding method proposed in this paper to construct threat exit variables can provide better explanatory effects in the deep learning model.

### 4.4 Robust tests

This paper adopts the following methods for robustness tests. First, we consider endogeneity tests. The industry mean for *Coz* and lagged three-period data for *Coz* are selected as instrumental variables. The two-stage least squares (2SLS) and Gaussian mixed model (GMM) are used to conduct instrumental variables tests on the above issues. The second is to replace the measure of the explanatory variable *PRECS*. *PRECS2* is calculated as the absolute value of the difference between the upper and lower limit of earnings forecast, divided by the total asset balance at the beginning of the year. When the value of *PRECS2* is equal to 0, the higher the precision and the higher the quality of management earnings forecast disclosure. The results in Tables 5 and 6 show that the results obtained from the two robustness tests are generally consistent with the results of the benchmark regression, with no significant changes in the coefficients and significance levels of the explanatory variables, making the benchmark regression results robust.

**Table 5:** Robustness test: IV regression between common institutional investors and management earnings forecast precision.

	(1)	(2)	(3)		(1)	(2)	(3)
	Step1-*Coz_num*	Step2-*PRECS*-2SLS	Step2-*PRECS*-GMM		Step1-*Coz_degree*	Step2-*PRECS*-2SLS	Step2-*PRECS*-GMM
m*Coz_num*ind	0.7863***			m*Coz_degree*ind	0.7574***		
	(0.1374)				(0.1412)		
L3*Coz_num*	0.4761***			L3*Coz_degree*	0.5695***		
	(0.0573)				(0.0622)		
*Coz_num*		-0.1144**	-0.1130**	*Coz_degree*		-0.0475**	-0.0473**
		(0.0533)	(0.0530)			(0.0221)	(0.0220)
*INSOWN*	0.1220***	-0.0624**	-0.0621**	*INSOWN*	0.2305***	-0.0653**	-0.0652**
	(0.0220)	(0.0262)	(0.0262)		(0.0418)	(0.0260)	(0.0260)
*BIGOWN1*	-0.1421***	0.0949**	0.0955**	*BIGOWN1*	-0.2921***	0.0971**	0.0973**
	(0.0340)	(0.0424)	(0.0424)		(0.0612)	(0.0422)	(0.0421)
*SIZE*	0.0255***	0.0074	0.0072	*SIZE*	0.0534***	0.0069	0.0068
	(0.0055)	(0.0066)	(0.0065)		(0.0106)	(0.0065)	(0.0064)
*LEV*	-0.1034***	0.0230	0.0237	*LEV*	-0.2164***	0.0251	0.0254
	(0.0249)	(0.0332)	(0.0331)		(0.0490)	(0.0330)	(0.0329)
*GSALES*	-0.0011	-0.0599***	-0.0601***	*GSALES*	0.0023	-0.0597***	-0.0597***
	(0.0095)	(0.0114)	(0.0114)		(0.0180)	(0.0114)	(0.0114)
*ROA*	-0.0023	-0.2282***	-0.2288***	*ROA*	-0.0297	-0.2302***	-0.2306***
	(0.0619)	(0.0860)	(0.0860)		(0.1160)	(0.0860)	(0.0860)
*OCF*	-0.0016	-0.1192	-0.1189	*OCF*	0.0385	-0.1164	-0.1161
	(0.0608)	(0.0785)	(0.0785)		(0.1109)	(0.0785)	(0.0784)
*DUAL*	-0.0092	0.0013	0.0014	*DUAL*	-0.0149	0.0017	0.0017
	(0.0082)	(0.0099)	(0.0099)		(0.0155)	(0.0099)	(0.0099)
*BRD*	0.0084	0.0316*	0.0315*	*BRD*	0.0016	0.0306	0.0305
	(0.0147)	(0.0189)	(0.0189)		(0.0274)	(0.0188)	(0.0188)
*BIG4*	0.1329***	0.0355	0.0359	*BIG4*	0.2920***	0.0342	0.0343
	(0.0346)	(0.0277)	(0.0277)		(0.0763)	(0.0276)	(0.0276)
*MGTSHR*	0.1105***	-0.0835**	-0.0833**	*MGTSHR*	0.1811***	-0.0875**	-0.0873**
	(0.0323)	(0.0380)	(0.0380)		(0.0563)	(0.0377)	(0.0377)
*PAY*	0.0052	0.0137*	0.0139*	*PAY*	0.0058	0.0135*	0.0135*
	(0.0067)	(0.0071)	(0.0071)		(0.0127)	(0.0071)	(0.0071)
*ANA*	0.0341***	-0.0266***	-0.0265***	*ANA*	0.0583***	-0.0277***	-0.0276***
	(0.0049)	(0.0051)	(0.0051)		(0.0093)	(0.0051)	(0.0051)
*VOL*	0.0099	-0.0378***	-0.0379***	*VOL*	0.0203	-0.0379***	-0.0380***
	(0.0080)	(0.0095)	(0.0095)		(0.0157)	(0.0095)	(0.0095)
_cons	-0.6897***	-0.2025	-0.1989	_cons	-1.3168***	-0.1856	-0.1844
	(0.1481)	(0.1724)	(0.1720)		(0.2801)	(0.1686)	(0.1684)
*Year* FE	Yes	Yes	Yes	Year FE	Yes	Yes	Yes
*Industry* FE	Yes	Yes	Yes	Industry FE	Yes	Yes	Yes
r2_a	0.3078	0.1180	0.1182	r2_a	0.3487	0.1206	0.1206
*N*	2291	2284	2284	*N*	2291	2284	2284
F		51.4435				56.2188	
*Hansen J statistic*		0.085036				0.015272	
*Pval of Hansen J statistic*		0.7706				0.9016	

Standard errors in parentheses

* *p* < 0.10

** *p* < 0.05

*** *p* < 0.01.

**Table 6:** Robustness test: Replacing the measure of earnings forecast precision.

	(1)	(2)	(3)	(4)
	*PRECS*2	*PRECS*2	*PRECS*2	*PRECS*2
*Coz_dum*	-0.0016***			
	(-3.5130)			
*Coz_num*		-0.0022***		
		(-3.5867)		
*Coz_degree*			-0.0013***	
			(-4.3377)	
*Coz_rate*				-0.0047***
				(-3.2559)
*INSOWN*	-0.0014*	-0.0014	-0.0014	-0.0016*
	(-1.6466)	(-1.6310)	(-1.5863)	(-1.8050)
*BIGOWN1*	0.0045***	0.0045***	0.0045***	0.0049***
	(3.5781)	(3.5757)	(3.5577)	(3.8420)
*SIZE*	-0.0015***	-0.0015***	-0.0015***	-0.0015***
	(-7.7003)	(-7.6634)	(-7.5891)	(-7.7476)
*LEV*	-0.0051***	-0.0051***	-0.0051***	-0.0051***
	(-5.0860)	(-5.0975)	(-5.1234)	(-5.0771)
*GSALES*	0.0025***	0.0025***	0.0025***	0.0025***
	(6.1264)	(6.1345)	(6.1116)	(6.1459)
*ROA*	0.0187***	0.0187***	0.0187***	0.0188***
	(4.0479)	(4.0523)	(4.0434)	(4.0534)
*OCF*	0.0163***	0.0163***	0.0163***	0.0162***
	(6.5644)	(6.5602)	(6.5883)	(6.5232)
*DUAL*	-0.0002	-0.0002	-0.0002	-0.0002
	(-0.5781)	(-0.5788)	(-0.5991)	(-0.5950)
*BRD*	0.0007	0.0007	0.0007	0.0007
	(1.3341)	(1.3459)	(1.3681)	(1.3365)
*BIG4*	-0.0007	-0.0007	-0.0007	-0.0008
	(-0.9174)	(-0.9026)	(-0.8356)	(-1.0501)
*MGTSHR*	0.0025*	0.0025*	0.0025*	0.0024*
	(1.8366)	(1.8461)	(1.8625)	(1.7703)
*PAY*	0.0014***	0.0014***	0.0014***	0.0014***
	(5.2102)	(5.2007)	(5.2345)	(5.1890)
*ANA*	0.0012***	0.0012***	0.0012***	0.0011***
	(6.9280)	(6.9163)	(6.8965)	(6.6965)
*VOL*	0.0013***	0.0013***	0.0013***	0.0013***
	(5.4214)	(5.4166)	(5.3932)	(5.3900)
_cons	0.0224***	0.0223***	0.0219***	0.0227***
	(4.4150)	(4.3952)	(4.3055)	(4.4866)
*Industry* FE	Yes	Yes	Yes	Yes
*Year* FE	Yes	Yes	Yes	Yes
*N*	8428	8428	8428	8428
r2_a	0.1830	0.1830	0.1836	0.1825

Note: T-values calculated from heteroskedasticity-robust standard errors and clustered at the firm level are in parentheses

(* p < 0.1, ** p < 0.05, *** p < 0.01.

## 5. Conclusion

The findings of this paper are as follows: Common institutional investors help to improve the precision of management’s earnings forecasts. It provides valuable insights into the role of institutional investors in shaping corporate decision-making and financial reporting practices, which has implications for investors, analysts, regulators, and corporate governance practitioners. Based on this, we propose the following suggestions: First, common institutional shareholders help to obtain more critical financial information from enterprises in the same industry, create a platform for information sharing, and reduce supervision costs for institutional participation in corporate governance due to their unique advantages in capital size, specific industry knowledge, and information collection and analysis. Secondly, listed companies should be encouraged to voluntarily disclose institutional shareholdings and give full play to information intermediaries’ monitoring and governance roles. Finally, the industry association should strengthen the communication between common institutional shareholders and the management of listed companies, further create an institutional environment for institutional investors to participate in corporate governance and play synergistic advantages, help improve and perfect the performance forecasts disclosure system, and realize mutual promotion and resource sharing among companies, investors and the industry.

## Supporting information

S1 File (ZIP)
